# Neurofilament-light chain quantification by Simoa and Ella in plasma from patients with dementia: a comparative study

**DOI:** 10.1038/s41598-023-29704-8

**Published:** 2023-03-10

**Authors:** Marta Truffi, Maria Garofalo, Alessandra Ricciardi, Matteo Cotta Ramusino, Giulia Perini, Silvia Scaranzin, Matteo Gastaldi, Sara Albasini, Alfredo Costa, Viola Chiavetta, Fabio Corsi, Carlo Morasso, Stella Gagliardi

**Affiliations:** 1 Istituti Clinici Scientifici Maugeri IRCCS, 27100 Pavia, Italy; 2 Molecular Biology and Transcriptomics Unit, IRCCS Mondino Foundation, 27100 Pavia, Italy; 3 Unit of Behavioral Neurology and Center for Cognitive Disorders and Dementia (CDCD), IRCCS Mondino Foundation, 27100 Pavia, Italy; 4 Department of Brain and Behavioral Sciences, University of Pavia, 27100 Pavia, Italy; 5 Neuroimmunology Laboratory, IRCCS Mondino Foundation, 27100 Pavia, Italy; 6 Department of Biomedical and Clinical Sciences, University of Milan, 20157 Milan, Italy

**Keywords:** Biological techniques, Neuroscience

## Abstract

Neurofilament light chains (NfL) are neuron-specific cytoskeletal proteins whose plasmatic concentrations have been explored as a clinically useful marker in several types of dementia. Plasma concentrations of NfL are extremely low, and just two assays are commercially available for their study: one based on the SiMoA technology and one based on Ella. We thus studied plasma levels of NfL with both platforms to check the correlation between them and to assess their potential in the diagnosis of neurodegeneration. Plasma NfL levels were measured on 50 subjects: 18 healthy controls, 20 Alzheimer’s disease, and 12 frontotemporal dementia patients. Ella returned plasmatic NfL levels significantly higher than SiMoA, however the results were strongly correlated (r = 0.94), and a proportional coefficient of 0.58 between the two assays was calculated. Both assays detected higher plasma NfL levels in patients with dementia than in the control group (*p* < 0.0001) and allowed their discrimination with excellent diagnostic performance (AUC > 0.95). No difference was found between Alzheimer’s and Frontotemporal dementia either using SiMoA or Ella. In conclusion, both the analytical platforms resulted effective in analysing plasma levels of NfL. However, the correct interpretation of results requires the precise knowledge of the assay used.

## Introduction

Neurofilament light chains (NfL) are important, neuron-specific cytoskeletal proteins present in the neurons’ bodies and axons, ensuring structural stability and axonal polarization^1^. Growing evidence indicates that NfL are non-specific markers of axonal damage, and their concentration is increased in the cerebrospinal fluid (CSF) and in the blood of patients with several neurodegenerative disorders. NfL were thus suggested as a circulating biomarker to discriminate between healthy controls and subjects with neurodegenerative condition^2,3^. In particular, increased CSF and blood NfL levels have been proposed as diagnostic and prognostic markers for dementia^4,5^. In fact, CSF and blood NfL levels are higher in patients with Alzheimer’s disease (AD)^6,7^. Furthermore, even if fewer data are available in the scientific literature on the topic, a similar pattern of significantly higher NfL levels in the CSF and serum of patients affected by frontotemporal dementia (FTD) was reported^8^.

NfL analysis is usually performed on CSF and serum, while plasma is less common. This is due to the presence in the plasma of blood-clotting agents that may interfere with laboratory tests. However, it has recently been demonstrated that plasma might be a preferable sample material for biomarker studies^9^. The reason for this lies in the fact that the processing of serum samples should be carefully checked because it may be risky to combine serum samples generated using different clotting procedures (e.g. initiated by thrombin vs. silicate-enhanced) into the same set of samples. On the contrary, plasma isolation depends only on centrifugation conditions. Furthermore, plasma samples are routinely collected in research laboratories and biobanks and are thus commonly available. It is also relevant to notice that many protocols for protein analysis based on plasma are considered valid for diagnostic use. Plasma levels of NfL are extremely low, in the low pg/mL range, and thus are undetectable to many of the traditional ELISA assays used in biomedical laboratories. The quantification of plasma NfL levels in clinics thus necessarily requires the use of new ultrasensitive techniques^10^.

At the time of the writing of this article, only two assays are commercially available for the study of plasma NfL: one based on the SiMoA technology and one based on Ella.

SiMoA is an assay for NfL detection from Quanterix. It is a digital immunoassay platform based on two highly specific non-competing monoclonal antibodies and arrays of femtoliter-sized microwells that can isolate and detect single molecules bound to paramagnetic beads. In the SiMoA each microbead is singularly entrapped in a microwell. Thus, the isolated beads are individually analyzed to reveal the presence or absence of the protein target on their surface, enhancing the sensitivity of traditional ELISA assays^11^. SiMoA makes use of a 96 wells plate format, but NfL are usually measured in triplicates on 25 subjects for each plate.

Ella is a microfluidic cartridge-based immunoassay platform from ProteinSimple (part of Bio-Techne), which is widely used to quantify soluble biomarkers^12,13^. Different cartridge formats allow measuring 72 samples in one assay. Measures are performed in triplicates in three glass nanoreactors (GNRs) for each sample using a fluorescent substrate, which helps to achieve a wide dynamic measuring range.

The ultrasensitive SiMoA technology was the first assay applied to establish serum NfL as a circulating biomarker in patients with multiple sclerosis^14^. As a result, it has become the premier solution for detecting NfL to advance the development of therapeutics and diagnostics in several neurodegenerative conditions, and a vast literature is now available on its use^15–20^.

The assay based on Ella has been introduced more recently and its performance in comparison with the one observed on SiMoA needs to be understood.

The sensitivity of the available Ella assays is in the lower pg/mL range with a required sample volume between 2.5 and 50 μL^12,13^. SiMoA reports an even lower sensitivity, below pg/mL concentrations^11^, and for the quantification of NfL requires 25 µL of plasma.

In a first study, Ella and SiMoA were compared in their ability to detect blood levels of NfL in serum samples of patients with multiple sclerosis^21^. As mentioned, in fact, in the literature NfL quantification is usually reported in serum and CSF, and the most cited method is the NfL assay by SiMoA^22–24^. Some data are also present on the Bio-Techne website^25^ on the comparison between Ella and a new NfL assay developed by Uman Diagnostic in the analysis of NfL in serum samples. To the best of our knowledge, at the date of the submission of this work, no data is still available on the comparison between the SiMoA and Ella NfL assays on plasma samples.

Given these considerations, we thus focused on investigating NfL levels in plasma samples of dementia, and matched controls, and we compared the results obtained using the two immunoassays. In particular, in this article, we focus on the quantification of NfL in plasma samples in two distinct dementia cohorts: AD, which is well characterized in terms of NfL quantification; and FTD patients, where only a few papers have been published^26,27^. The results obtained will thus simplify the translation towards the clinical use of NfL in dementia by providing helpful information on NfL levels in a biofluid widely available, such as plasma; and by reporting the first publicly available data on the relationship between plasma NfL levels measured in parallel by SiMoA and Ella.

## Results

### Comparison between SiMoA and Ella for plasma NfL quantification

**Figure 1:**
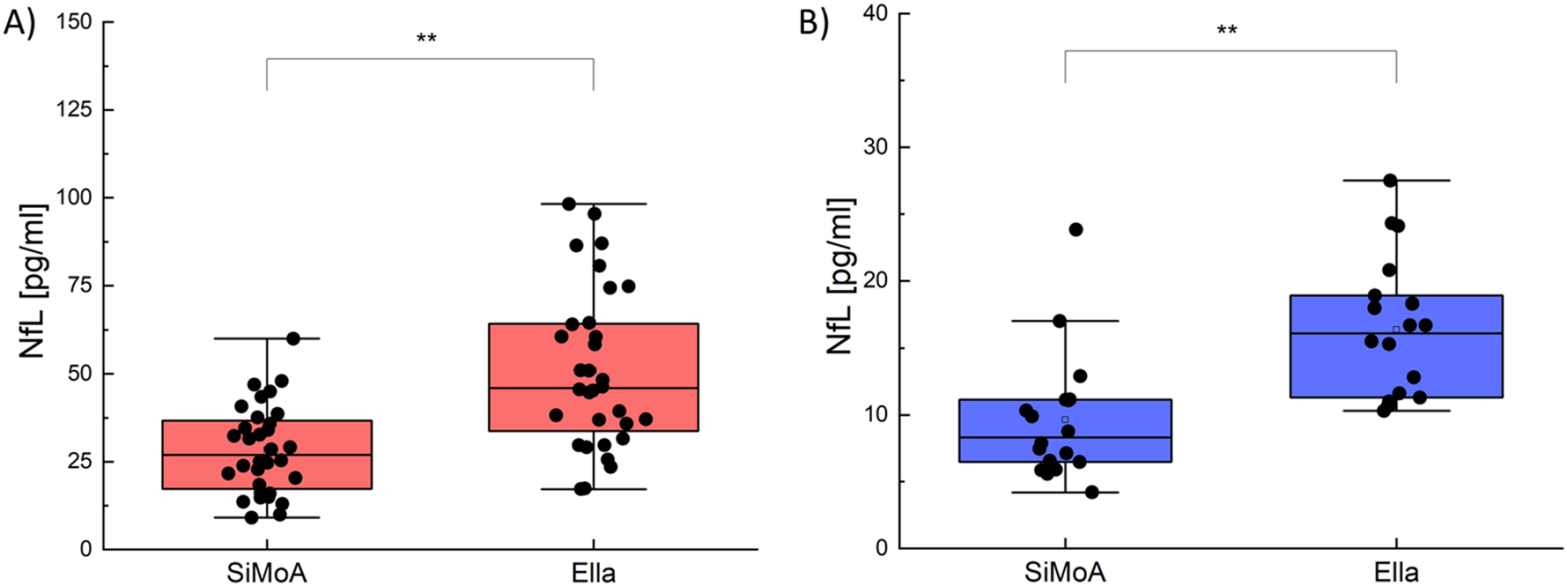
(**A**) Plasma levels of NfL measured by SiMoA and Ella in patients with dementia (AD plus FTD). (**B**) Plasma levels of NfL measured by SiMoA and Ella in HC. Each data point represents an individual subject analysed. Each box represents the area between the 25th and 75th percentiles [interquartile range, IQR]. Lines inside the boxes represent the median values. White dots represent the mean value for each class. Whiskers extend to the lowest and highest values within 1.5 times the IQR from the box.

To make a direct comparison between the performances of the SiMoA and Ella technologies in assessing plasma NfL concentration in patients with dementia, anonymized human samples from a cohort of 50 subjects was analysed in parallel. Both SiMoA and Ella showed optimal repeatability, with the mean intra-assay coefficient of variation (CV) on Ella technology equal to 5.14% (± 3.12) vs. 5.88% (± 3.86) on SiMoA (*p* = 0.27) (see Supplementary Table S1 Supplementary Fig. S1). In a cohort of 32 patients with dementia, including 20 patients with AD and 12 with FTD, the mean plasma NfL concentration measured by Ella was significantly higher than the one obtained by SiMoA (MED: 45.95 IQR: 30.60 pg/mL vs. MED: 26.95 IQR: 19.45 pg/mL; *p* < 0.0001) (Fig. 1A). Similarly, in the healthy controls (HC) cohort, Ella measured higher NfL levels as compared to SiMoA (MED: 16.10 IQR: 7.60 pg/mL vs. MED: 8.31 IQR: 4.67 pg/mL; *p* = 0.0016) (Fig. 1B). In both dementia and HC groups, the distribution of NfL values more scattered in Ella than SiMoA. Overall, NfL levels measured with Ella showed higher standard deviation (SD) than those measured by SiMoA (SD = 22.6 vs. SD = 12.5 respectively, Levene test *p* = 0.002). This difference was leaded by dementia group, in fact measures’ SD with Ella was higher than with SiMoA (SD = 24.7 vs. SD = 13.8 respectively, Levene test *p* = 0.0002) but there was no statistical difference in HC group (SD = 5.3 vs. SD = 4.7 respectively, Levene test *p* = 0.42).

**Figure 2:**
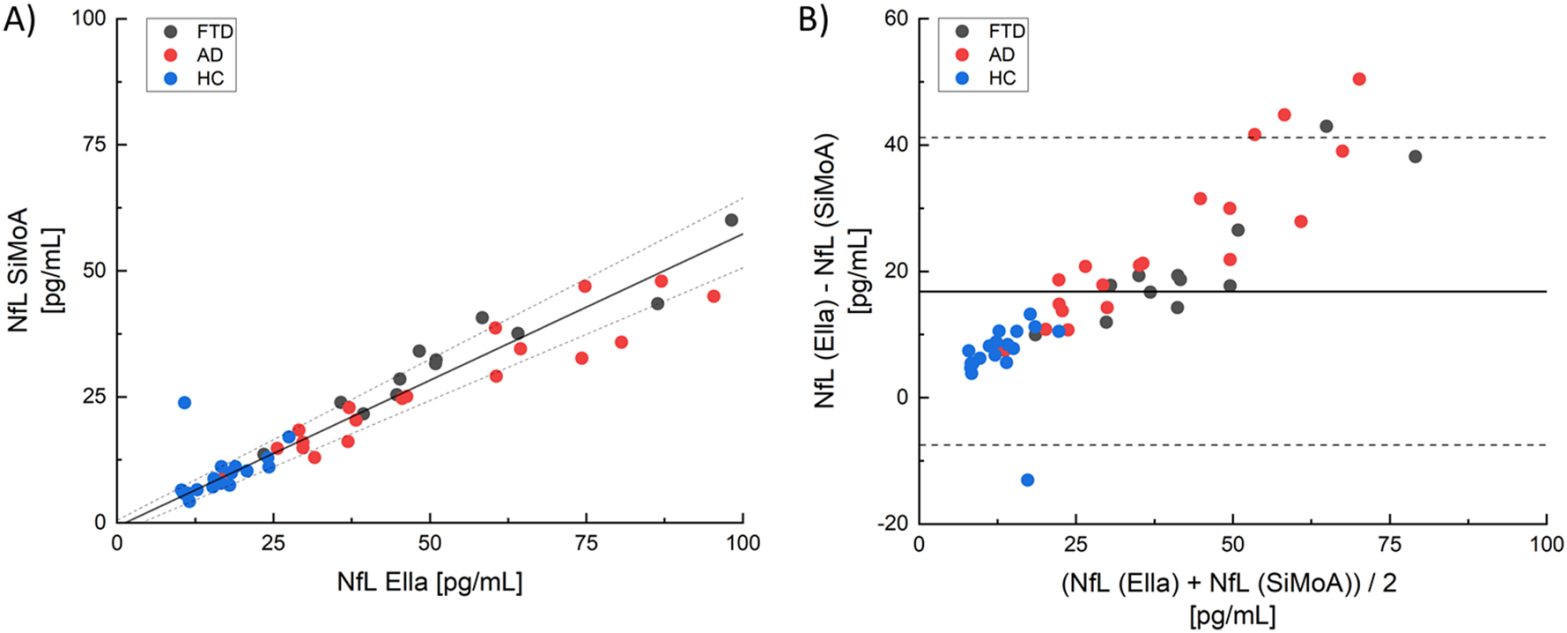
(**A**) Passing–Bablok regression analysis of NfL concentration measured by Ella and SiMoA. Solid black line: Passing–Bablok regression fit. The dashed lines represent 95% limits of agreement. (**B**) Bland–Altman plots comparing the agreement between plasma NfL levels measured by Ella and SiMoA. The solid line represents the difference between the assays. The dashed lines represent 95% limits of agreement. Each data point represents an individual subject analysed (Blue: HC; Red: AD; Dark grey: FTD).

Plasma NfL levels in the entire study population were strongly correlated between the two technologies (Spearman r = 0.94; *p* < 0.0001). The Passing Bablock regression depicted a linear dependence between the two datasets (Fig. 2A). The slope of the regression line was 0.58, confirming that Ella measurements were higher and did not overlap with those obtained by SiMoA in matched observations. The Bland Altman method further confirmed an agreement between the two assays (Fig. 2B). Globally, 90% of observations were within the limit of agreement as assessed by the confidence lines (displayed as dashed lines in the graph). The Bland–Altman method displayed a bias of 16.8% between the NfL concentrations obtained with the two technologies; hence Ella showed a mean 16.8% overestimation as compared to SiMoA. It has to be noted that the observations made with Ella and SiMoA were more dispersed when NfL values, calculated as average of Ella and SiMoA techniques, were higher (Fig. 2B). Therefore, the difference between Ella and SiMoA methods increased when the levels of NfL increased.

### Plasma NfL in Alzheimer’s disease and frontotemporal dementia

**Figure 3:**
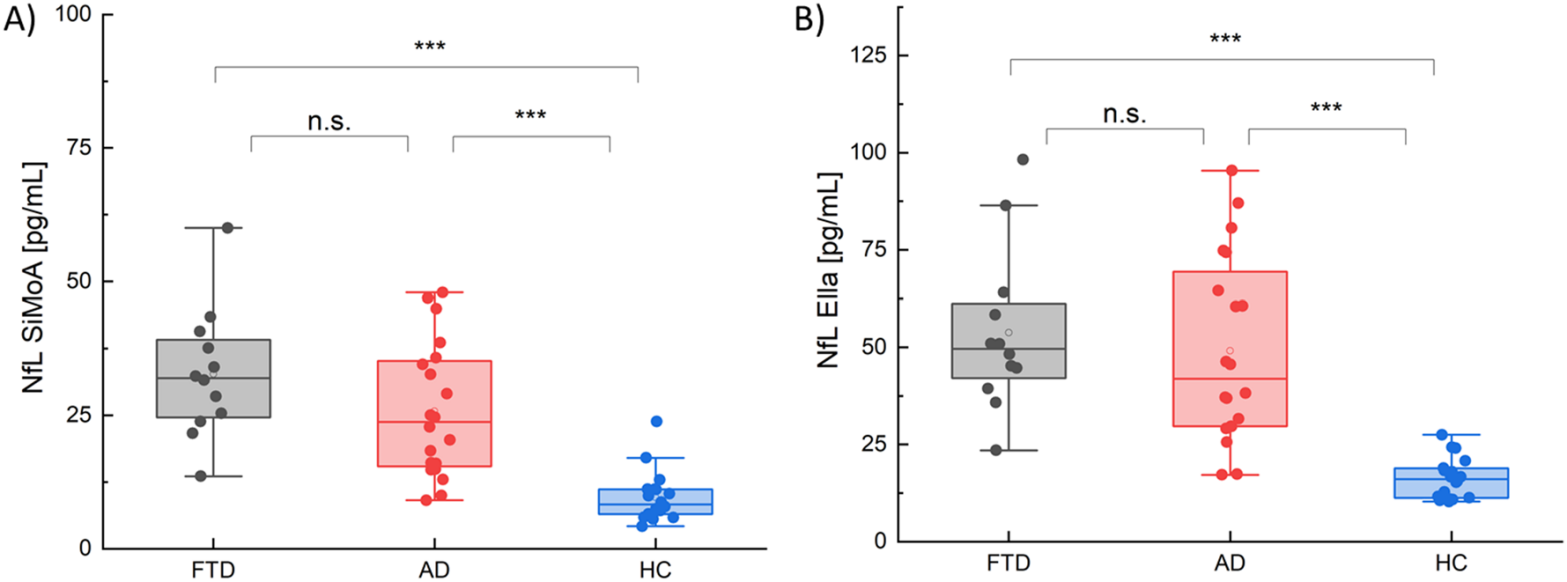
(**A**) Plasma levels of NfL measured by SiMoA in FTD, AD and HC. (**B**) Plasma levels of NfL measured by Ella in FTD, AD and HC. Each data point represents an individual subject analysed. Each box represents the area between the 25th and 75th percentiles [interquartile range, IQR]. Lines inside the boxes represent the median values. White dots represent the mean value for each class. Whiskers extend to the lowest and highest values within 1.5 times the IQR from the box.

Plasma levels of NfL measured both by SiMoA and Ella were compared between AD, FTD and HC (Fig. 3). The comparison took place two-by-two between groups. When measured by SiMoA, both AD and FTD groups presented higher plasma NfL levels when compared with HC (AD vs. HC *p* < 0.0001; FTD vs. HC *p* < 0.0001). No statistically significant difference was detected between AD (MED: 23.74 IQR: 19.75 pg/mL) and FTD patients (MED: 31.95 IQR: 14.51 pg/mL; *p* = 0.18) (Fig. 3A). Globally, plasma NfL levels were found to be higher in patients with a dementia (AD and FTD together) (MED: 26.95 IQR: 19.45 pg/mL) when compared with the HC (MED: 8.31 IQR: 4.67 pg/mL; *p* < 0.0001; AUC = 0.95). A cut-off value between the two populations was calculated with a ROC curve at 12.95 pg/mL (see Supplementary Fig. S2) in order to distinguish two groups with a reference NfL level. Similarly, when measured by Ella, both AD and FTD groups presented higher plasma NfL levels when compared with HC (AD vs. HC *p* < 0.0001; FTD vs. HC *p* < 0.0001). No statistically significant difference was detected between AD (MED: 41.90 IQR: 39.70 pg/mL) and FTD patients (MED: 49.60 IQR: 19.20 pg/mL; *p* = 0.48) (Fig. 3B). Plasma NfL levels were found to be higher in patients with a dementia (AD and FTD together) (MED: 45.95 IQR: 30.60 pg/mL) when compared with the control HC (MED: 16.10 IQR: 7.60 pg/mL; *p* < 0.0001; AUC = 0.97). A cut-off value between the two population was calculated with a ROC curve at 25.60 pg/mL (see Supplementary Fig. S3).

## Discussion

Circulating NfL levels are a biomarker of great interest in the neurological field. Their measure provides an objective way to detect neuronal damage through a simple blood analysis that is much easier to perform than a CSF collection or an imaging-based approach (MRI or PET). Therefore, they can support clinical neurologists in the diagnosis in the early phases of neurodegenerative diseases so that patients can receive prompt treatment. A vast literature now proves that circulating NfL levels are higher in patients with a different kind of dementia with respect to healthy subjects of similar ages^28,29^. While blood levels of NfL cannot discriminate between subjects with different neurological conditions, they can differentiate patients with a different degree of disease severity^30^.

The quantification of circulating NfL levels is most usually reported using an ultrasensitive assay developed by Quanterix and based on the use of the SiMoA technology. More recently, a second NfL assay based on the Ella technology by ProteinSimple was released. At the date of the writing process of this manuscript, none of these two assays has been approved by the Food and Drug Administration for clinical use. However, blood NfL levels are now commonly measured in many clinical research projects on dementia.

Given the importance that NfL might have in the field, in this work, we performed a parallel measure of NfL in the same plasma sample of patients with neurodegenerative dementia and in a control group of healthy subjects using both the SiMoA and Ella assays.

The results obtained showed a very high level of correlation with a calculated rank correlation coefficient of 0.94. This result proves that, in principle, both approaches are suitable for the task and could be used in clinical studies to gain information on the levels of this circulating biomarker. Nevertheless, it is essential to notice that the absolute levels of NfL detected are different when the two approaches are used. The results obtained with Ella are significantly higher than the ones obtained using the SiMoA with a calculated proportional coefficient of 0.58 (NfL (Ella) * 0.58 = NfL (SiMoA)). Besides, data obtained from Ella have a higher variability, although the exact reason for this is currently not known.

These results generally confirm what was reported in a previous study by the Observatoire Français de la Sclérose en Plaques (OFSEP)^21^, where serum levels of NfL were measured using Ella and SiMoA on 203 patients with multiple sclerosis. Even in that case, a linear correlation was found between the two assays. However, the coefficient of proportionality in serum was found to be 0.34 with an even higher discrepancy between the absolute values. This difference in the slope of the Passing–Bablok regression fit between this work and the OFSEP article highlights a different effect of the two matrices used (plasma in this work, serum for OFSEP) on the two assays. Furthermore, attention must be given to the fact that in the Bland–Altman plots (Fig. 2B) the patients outside the 95% limits of agreement are concentrated in the group with a high plasma NfL level. This suggests that the linearity between the two assays might be lost at high concentrations, although further studies are needed to support this hypothesis.

The interpretation of the plasma NfL levels thus requires the precise knowledge of the assay used for the analysis. It is notable to observe how the median value of NfL detected by Ella in the HC group is above the calculated optimal cut-off between the patients affected by dementia and HC in the analysis performed using the SiMoA assay. The lack of specification of the assay used for the analysis could thus result in a misinterpretation of the data.

Regarding the clinical meaning of plasma NfL levels, high plasma NfL was found in both AD and FTD compared to controls independently from the technology used, making NfL a valuable neurodegeneration marker. However, no differences were found between the two types of dementia considered. While the HC group is slightly younger than AD and FTD, it has been reported in the literature that NfL levels may correlate with age, but mainly in subjects > 70 years old and associated with comorbidities, such as renal dysfunction and high body mass index (BMI)^31^. The cohort od AD patients included in our study has an inverted ratio of male/female subjects included, however the sex of the subjects do not affect the blood concentration of NfL and thus should not affect our results^31^. Furthermore, the prevalence of female patients in our AD cohort is justified by the clinical presentation of the disease that has a higher incidence in women^32^.

In addition, Ashton and collaborators showed that plasma tube type may also influence the NfL quantification^33^. In their work, Ashton and collaborators showed that the citrate tube type (used in the present study) had the lowest mean concentration for NfL, but this does not weaken results as we detected a marked difference between patients and HC.

Nevertheless, the significant difference observed between AD and FTD patients compared to controls may allow the identification of a “conservative” cut-off, a threshold below which only healthy controls are likely to be.

In conclusion, plasma NfL quantification may be considered a reliable biomarker that is not influenced by the method of detection used. This result confirms NfL importance in neurodegenerative diseases. Unfortunately, while NfL may be considered a marker of neurodegeneration, they are not disease-specific. Multiple reports in the scientific literature show that, as in AD and FTD, high plasmatic levels of NfL can be found in other neurological disorders, such as amyotrophic lateral sclerosis, multiple sclerosis^34^, and spinal cord injury^35^.

## Methods

### Study subjects

FTD and AD patients were recruited at the IRCCS Mondino Foundation, Pavia (Italy). Diagnosis of FTD was based on Rascovsky criteria^36^, while diagnosis of AD was based on criteria of the National Institute on Aging-Alzheimer’s Association (NIA-AA)^37^. The control subjects were recruited at the Transfusional Service and Centre of Transplantation Immunology, IRCCS Foundation San Matteo, Pavia (Italy). Only subjects not affected by any neurological condition or other relevant comorbidities were selected as HC.

Plasma from 18 HC, 12 FTD and 20 AD patients were collected. Baseline characteristics of study subjects are reported in Table 1.

**Table 1:** Baseline characteristics of recruited subjects for this study. HC = healthy controls; FTD = Frontotemporal Dementia; AD = Alzheimer’s Disease.

	HC	FTD	AD	*p*-value
Recruited subjects	18	12	20	
Age (mean ± SD)	60.4 ± 7.1	67.3 ± 5.5	71.5 ± 12.1	0.0004
Males %	61%	67%	30%	0.08
Females %	39%	33%	70%	

### Plasma collection

Venous blood (15 mL) was collected in sodium citrate tubes from FTD and AD patients, and HC. Blood was then centrifuged at 1000×*g* for 15 min, the obtained plasma was transferred into new 1.5 mL tubes and centrifuged at 1600×*g* for 20 min to remove platelets. Platelet-poor plasma was again transferred into 1.5 mL tubes and stored at − 80 °C until use.

### NfL assay (SiMoA)

NfL concentration was measured using a commercially available NF-light™ Advantage Kit (Item 103,400) for the Single Molecule Array (SiMoA) immunoassay SR-X (Quanterix, Lexington, MA, USA). Analysis was carried out using a 2-step assay following the manufacturer’s instructions. Calibrators points were run in duplicates while samples were analyzed in triplicates with a dilution of 1:4. The lower limit of quantification (LLOQ) of the NF-L assay was 0.316 pg/mL, and the limit of detection (LOD) was 0.0552 pg/mL. The intra-assay CV of replicates were automatically calculated by the instrument to assess the repeatability of the test, and expressed as percentages.

### NfL assay (Ella)

Human NF-L Simple Plex assay (ProteinSimple, CA, USA) was employed on Ella device (ProteinSimple, CA, USA), according to the manufacturers’ instructions. Calibration of Ella was performed using the incartridge factory standard curve, and plasma samples were measured with a 1:2 dilution in Sample Diluent (ProteinSimple, CA, USA). A single well was used for each sample as triplicates assays are automatically performed in Simple Plex assay microfluidic platform. The LLOQ was 2.70 pg/ml; the upper limit of quantification was 10,290.00 pg/ml.

### Statistical analysis

The sample size was calculated to determine whether the correlation coefficient differed from zero with a confidence level of 95% and a power of 80%. In particular, with a total of 50 patients, we can consider an expected correlation coefficient greater than 0.40 (moderate correlation).

The non-normal distribution of the NfL measurements obtained with SiMoA and Ella platforms was assessed by Shapiro–Wilk test. Spearman correlation coefficient was calculated to assess the association between plasma NfL concentrations obtained by SiMoA and Ella on the same sample series, and presented with confidence interval of 95%. Agreement between SiMoA and Ella technologies was measured by Passing Bablock regression and Bland–Altman methods. The Levene test was conducted to evaluate the differences in variance obtained by SiMoA and Ella technologies.

The mean NfL values obtained in AD, FTD and HC groups were compared using the non-parametric Wilcoxon test (or Kruskal–Wallis test in case of comparison between more than 2 category). Categorical variables were compared using Fisher exact test. Statistical significance was set at *p*-value < 0.05. Optimal NfL cutoffs, distinguishing HC controls among diseased sample, were determined by a Receiver Operating Characteristic (ROC) analysis for both SiMoA and Ella measurements.

Statistical analysis was performed using OriginLab, R software (v. 3.5.1, © The R Foundation) and SAS software [v. 9.4 SAS Institute Inc., Cary, USA].

### Informed consent

All patients included in the study signed an informed consent approved by the local ethics committee of San Matteo Hospital Foundation, Pavia (http://www.sanmatteo.org/site/home/attivita-scientifica/sperimentazioni-cliniche/comitato-etico.html) (for patients with FTD, Protocol n-20180049077; for patients with AD, Protocol n-20170016071; for HC subjects, Protocol n-20200045845, entire study protocol was approved by Protocol n-20220031286). All experiments were performed in accordance with relevant guidelines and regulations.

## Supplementary Information

Supplementary Information.

## Data Availability

Raw data are available by corresponding authors on request.
