# Supplementary material for: Neurofilament-light chain quantification by Simoa and Ella in plasma from patients with dementia: a comparative study
Source: Sci Rep. 2023 Mar 10;13:4041. doi: 10.1038/s41598-023-29704-8 (PMC10006166; doi:10.1038/s41598-023-29704-8)
Supplement: Supplementary file 1 — Supplementary Information. [file 41598_2023_29704_MOESM1_ESM.docx]

**Neurofilament-light chain quantification by Simoa and Ella in plasma from patients with dementia: a comparative study**

**Marta Truffi^1+^, Maria Garofalo^2+^, Alessandra Ricciardi^1^, Matteo Cotta Ramusino^3^, Giulia Perini^3,4^, Silvia Scaranzin^5^, Matteo Gastaldi^5^, Sara Albasini^1^, Alfredo Costa^3,4^, Viola Chiavetta^1^, Fabio Corsi^1,6^, Carlo Morasso^1^°*, Stella Gagliardi^2^°***

1 Istituti Clinici Scientifici Maugeri IRCCS Spa SB, Pavia, 27100, Italy

2 IRCCS Mondino Foundation, Molecular Biology and Transcriptomics Unit, Pavia, 27100, Italy

3 IRCCS Mondino Foundation, Unit of Behavioral Neurology and Center for Cognitive Disorders and Dementia (CDCD), Pavia, 27100, Italy

4 University of Pavia, Department of Brain and Behavioral Sciences, Pavia, 27100, Italy

5 IRCCS Mondino Foundation, Neuroimmunology Laboratory, Pavia, 27100, Italy

6 University of Milan, Department of Biomedical and Clinical Sciences, Milan, 20157, Italy

* corresponding authors: Stella Gagliardi, [stella.gagliardi@mondino.it](mailto:stella.gagliardi@mondino.it); Carlo Morasso, [carlo.morasso@icsmaugeri.it](mailto:carlo.morasso@icsmaugeri.it)

+,**_°_** these authors contributed equally to this work

**SUPPLEMENTARY INFORMATION**

**Supplementary Figure S1.** Boxplot showing the coefficient of variation (CV) for the NfL results measured by SiMoA and Ella in the study population (n = 50).

**
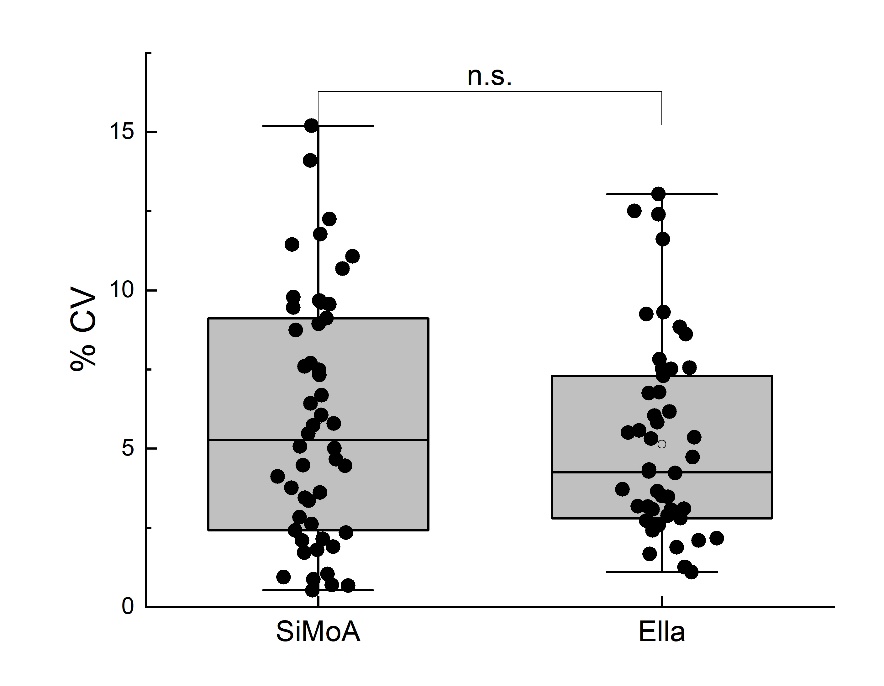
**

**Supplementary Figure S2.** Receiver operating characteristic (ROC) curve for NfL level’s accuracy to distinguish patients with dementia from healthy controls by means of SiMoA.


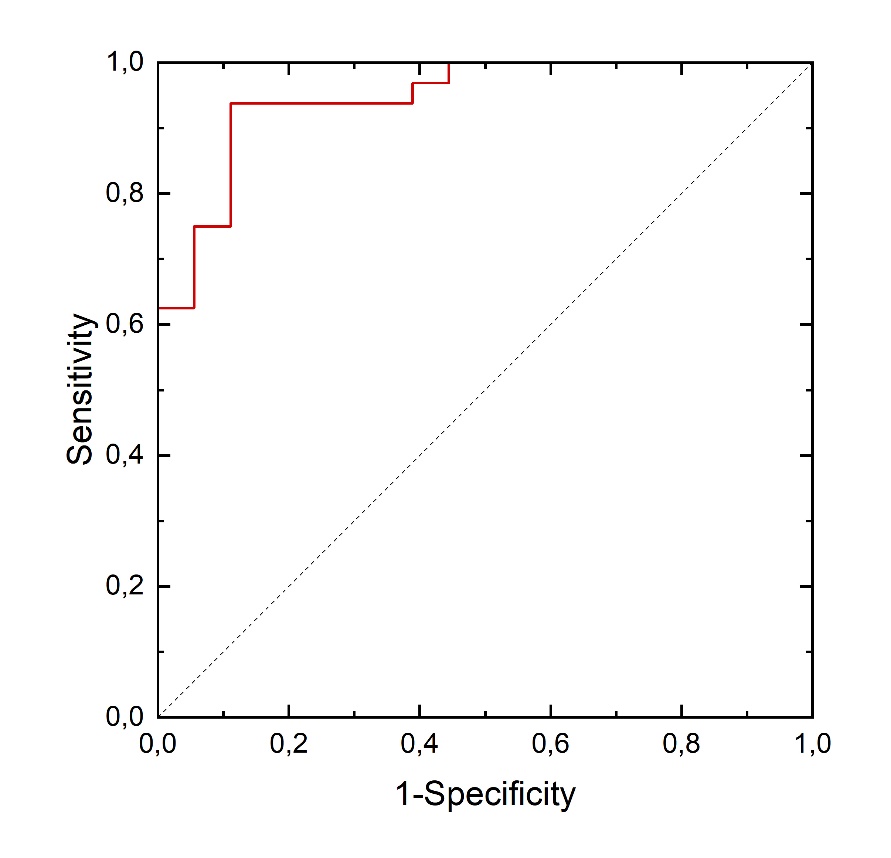


**Supplementary Figure S3.** Receiver operating characteristic (ROC) curve for NfL level’s accuracy to distinguish patients with dementia from healthy controls by means of Ella.


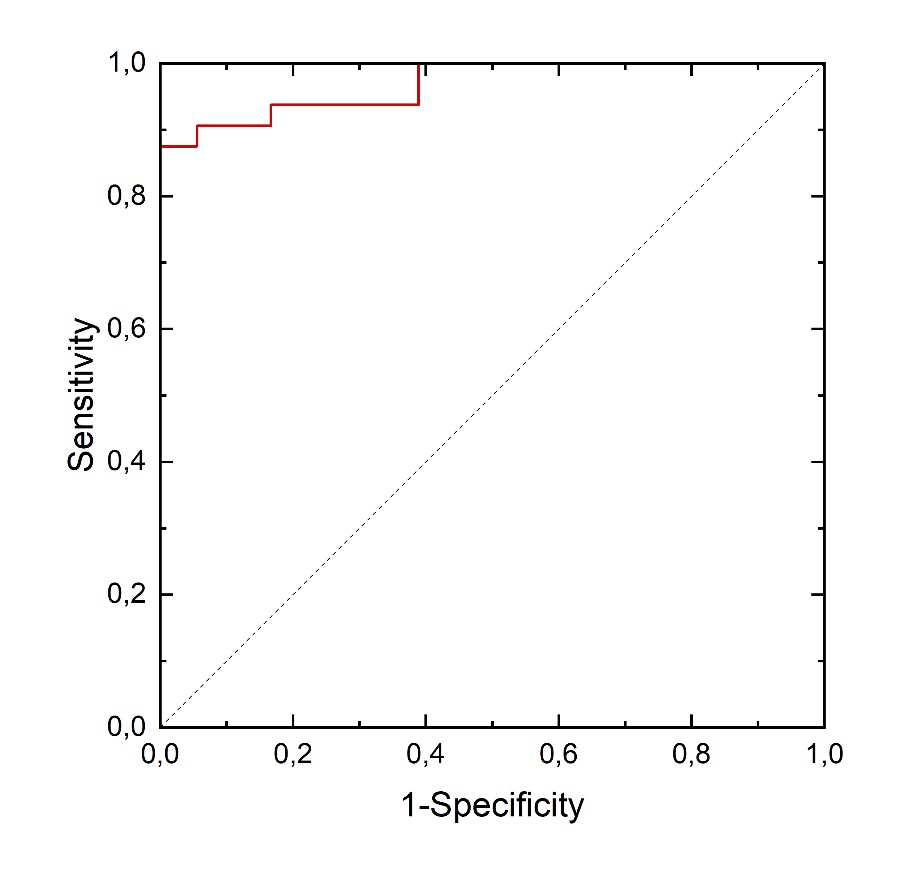


**Supplementary Table S1.** NfL Mean Concentration [pg/ml] and CV [%] obtained by SiMoA and Ella platform.

| **SAMPLES** | **AGE** | **GENDER** | **NF-L Mean Conc SiMoA [pg/ml]** | **%CV** | **NF-L Mean Conc ELLA [pg/ml]** | **%CV** |
| --- | --- | --- | --- | --- | --- | --- |
| **FTD1** | **78** | **M** | **13.58** | **9.78** | **23.50** | **3.08** |
| **FTD2** | **67** | **M** | **37.58** | **5.48** | **64.10** | **2.17** |
| **FTD3** | **64** | **F** | **31.58** | **2.83** | **50.90** | **6.04** |
| **FTD4** | **63** | **F** | **60.03** | **2.35** | **98.20** | **3.47** |
| **FTD5** | **72** | **M** | **32.32** | **0.93** | **51.00** | **4.73** |
| **FTD6** | **68** | **M** | **28.52** | **2.14** | **45.20** | **3.71** |
| **FTD7** | **65** | **M** | **43.45** | **0.53** | **86.40** | **1.88** |
| **FTD8** | **62** | **F** | **40.69** | **3.76** | **58.40** | **3.65** |
| **FTD9** | **70** | **M** | **34.04** | **9.67** | **48.30** | **3.07** |
| **FTD10** | **74** | **M** | **25.39** | **1.90** | **44.70** | **2.10** |
| **FTD11** | **58** | **M** | **23.85** | **9.46** | **35.80** | **5.50** |
| **FTD12** | **67** | **F** | **21.63** | **4.12** | **39.40** | **2.94** |
| **AD1** | **77** | **F** | **38.62** | **9.56** | **60.50** | **2.88** |
| **AD2** | **80** | **F** | **47.96** | **5.79** | **87.00** | **2.72** |
| **AD3** | **51** | **F** | **9.09** | **2.10** | **17.20** | **8.61** |
| **AD4** | **69** | **F** | **24.65** | **8.94** | **45.60** | **2.80** |
| **AD5** | **73** | **M** | **44.95** | **6.68** | **95.40** | **1.67** |
| **AD6** | **70** | **M** | **15.95** | **6.06** | **29.70** | **3.10** |
| **AD7** | **57** | **M** | **9.95** | **0.69** | **17.40** | **4.34** |
| **AD8** | **84** | **M** | **22.84** | **3.35** | **37.10** | **1.26** |
| **AD9** | **78** | **F** | **18.37** | **14.10** | **29.10** | **3.50** |
| **AD10** | **81** | **F** | **35.81** | **9.61** | **80.60** | **3.17** |
| **AD11** | **35** | **F** | **12.95** | **5.00** | **31.60** | **2.58** |
| **AD12** | **77** | **F** | **20.37** | **0.67** | **38.20** | **7.51** |
| **AD13** | **76** | **M** | **14.77** | **2.61** | **25.60** | **5.32** |
| **AD14** | **77** | **M** | **34.54** | **2.42** | **64.50** | **1.25** |
| **AD15** | **69** | **F** | **25.05** | **7.70** | **46.30** | **8.84** |
| **AD16** | **85** | **F** | **46.92** | **3.44** | **74.80** | **4.28** |
| **AD17** | **73** | **F** | **29.07** | **10.69** | **60.60** | **6.17** |
| **AD18** | **82** | **F** | **32.67** | **6.43** | **74.30** | **2.56** |
| **AD19** | **69** | **F** | **16.11** | **15.20** | **36.90** | **7.52** |
| **AD20** | **67** | **F** | **14.90** | **3.62** | **29.70** | **7.82** |
| **HC1** | **47** | **F** | **7.86** | **4.67** | **16.70** | **5.36** |
| **HC2** | **54** | **M** | **5.91** | **8.75** | **10.60** | **5.83** |
| **HC3** | **55** | **M** | **5.59** | **5.73** | **11.00** | **7.55** |
| **HC4** | **46** | **F** | **4.20** | **11.45** | **11.60** | **12.40** |
| **HC5** | **69** | **M** | **9.90** | **1.71** | **18.30** | **2.41** |
| **HC6** | **70** | **M** | **11.16** | **1.80** | **16.70** | **4.23** |
| **HC7** | **67** | **F** | **17.02** | **5.07** | **27.50** | **9.31** |
| **HC8** | **69** | **M** | **7.46** | **7.32** | **18.00** | **6.75** |
| **HC9** | **59** | **F** | **7.13** | **1.03** | **15.30** | **13.04** |
| **HC10** | **60** | **M** | **6.47** | **0.87** | **10.30** | **1.10** |
| **HC11** | **64** | **M** | **5.86** | **11.07** | **11.30** | **3.18** |
| **HC12** | **60** | **F** | **10.31** | **4.45** | **20.80** | **6.78** |
| **HC13** | **60** | **M** | **23.84** | **11.77** | **10.80** | **12.51** |
| **HC14** | **65** | **M** | **12.90** | **9.12** | **24.10** | **11.61** |
| **HC15** | **55** | **F** | **6.55** | **7.60** | **12.80** | **7.30** |
| **HC16** | **67** | **M** | **11.14** | **4.48** | **18.90** | **5.57** |
| **HC17** | **61** | **M** | **11.09** | **7.48** | **24.30** | **2.58** |
| **HC18** | **59** | **F** | **8.76** | **12.25** | **15.50** | **9.25** |
